# Supplementary material for: PNPLA1 has a crucial role in skin barrier function by directing acylceramide biosynthesis
Source: Nat Commun. 2017 Mar 1;8:14609. doi: 10.1038/ncomms14609 (PMC5337976; doi:10.1038/ncomms14609)
Supplement: Supplementary Information — Supplementary Figures and Supplementary Tables. [file ncomms14609-s1.pdf]

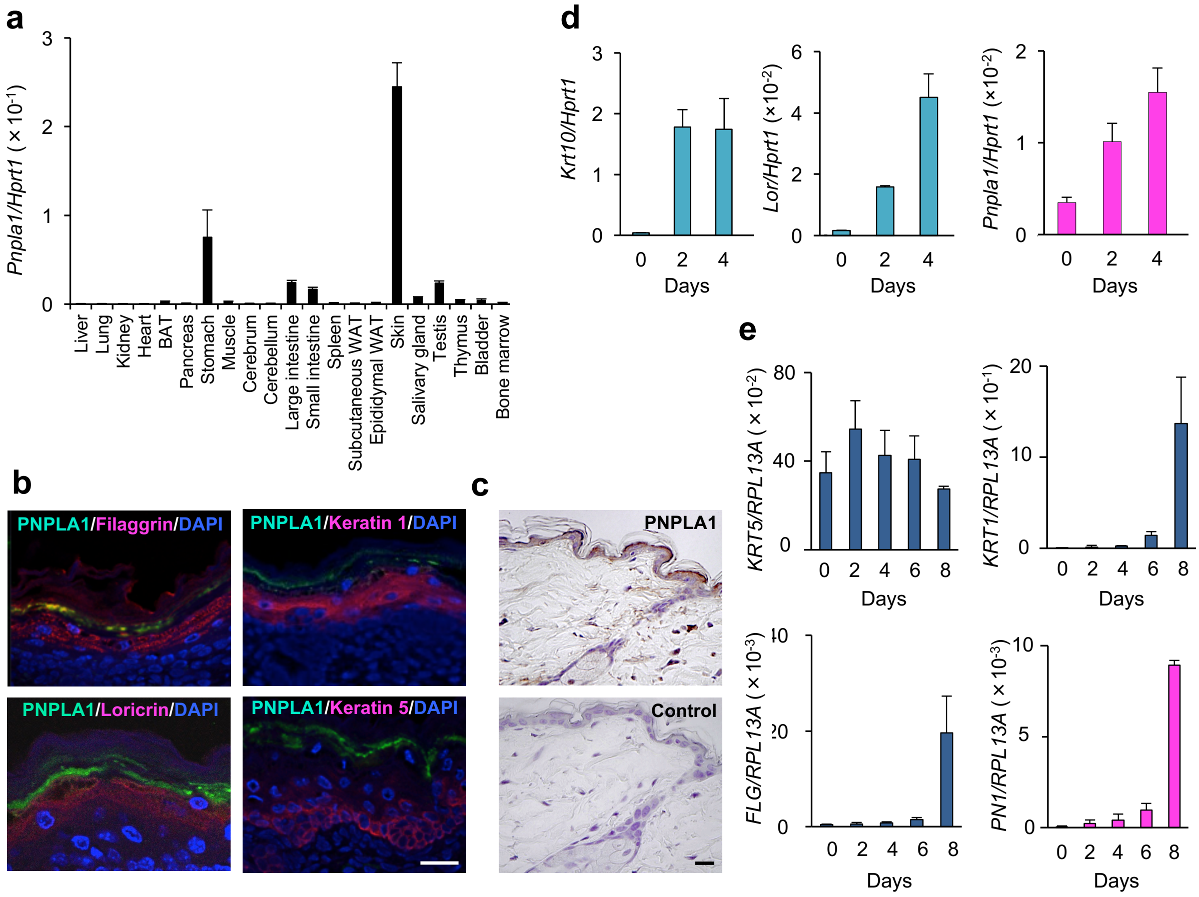

Supplementary Fig. 1. ***Pnpla1*** **expression in the skin and differentiated cultured keratinocytes.**
(**a**) qPCR analysis of *Pnpla1* expression in various tissues of 8-week-old male C57BL/6 mice, with *Hprt1* as an internal control (mean ± s.e.m., n = 6 animals). (**b**) Immunofluorescent staining of newborn mouse skin sections with antibodies specific for PNPLA1 (green) and filaggrin, loricrin, keratin 1 or keratin 5 (red). Nuclei were counterstained with DAPI (blue). (**c**) Immunohistochemical staining of the back skin sections of adult C57BL/6 mice (8-week-old) with anti-PNPLA1 antibody (upper panel) or isotype-matched control antibody (lower panel). The sections were counterstained with hematoxylin. (**d, e**) qPCR analysis of expression levels of *Pnpla1*/*PNPLA1* and keratinocyte differentiation markers in mouse (**d**) and human (**e**) keratinocytes in culture with 1.2 mM $CaCl_2$ for the indicated periods, with *Hprt1* and *RPL13A,* respectively, as internal controls (mean ± s.e.m., n = 3). Results are representative of two or three independent experiments. Scale bar, 20 μm.

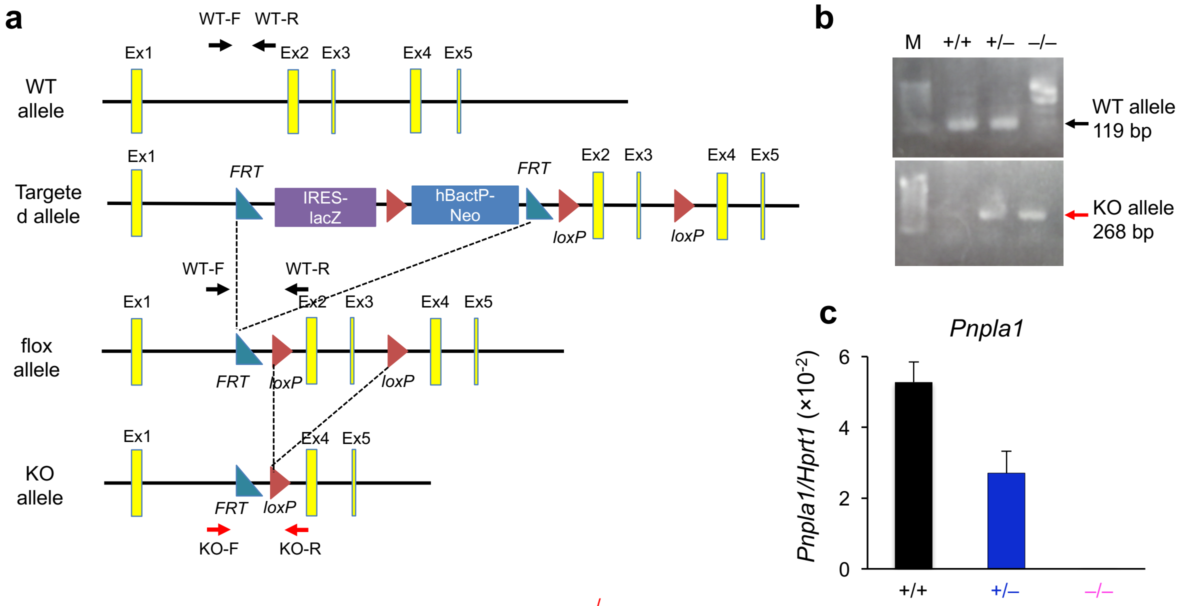

d

+/+	+/–	–/–	Total
51	94	45	190

e

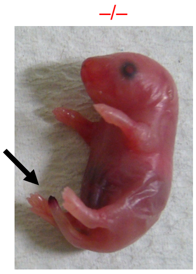

Supplementary Fig. 2. Generation of *Pnpla1*-deficient mice.
(a) Strategy of *Pnpla1* gene targeting. In the targeted allele, exons 2 and 3 were flanked by *loxP* sites, and the *IRES-LacZ* and *Neo* cassettes flanked by *FRT* sites were inserted between exons 1 and 2. *FRT* and *loxP* sites are depicted as blue and red triangles, respectively. The positions of primers for genotyping PCR (arrows labeled WT-F, WT-R, KO-F, and KO-R) are indicated. *Neo*, neomycin phosphotransferase gene. **(b)** PCR genotyping of tail DNA, amplifying 119-bp and 268-bp products of WT and KO alleles, respectively. **(c)** qPCR analysis of *Pnpla1* mRNA expression in the skin of $Pnpla1^{+/+}$, $Pnpla1^{+/-}$ and $Pnpla1^{-/-}$ mice at P0 (mean ± s.e.m., n = 5 animals per group). **(d)** Frequencies of *Pnpla1* genotypes in pups derived from intercrosses between heterozygous mice. **(e)** Tail tip necrosis (arrow) in $Pnpla1^{-/-}$ newborn mice. Representative (**b, c, e**) and compiled (**d**) results from multiple experiments are shown.

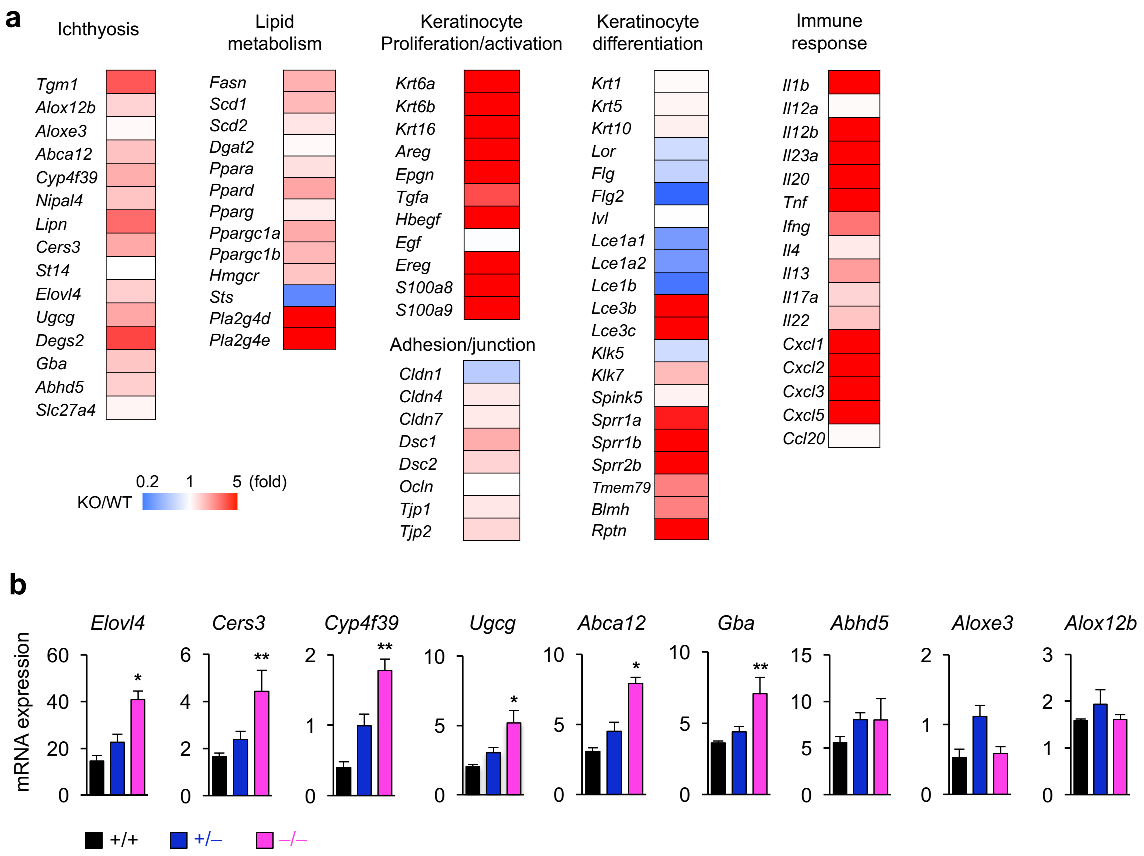

Supplementary Fig. 3. Changes in gene expression in *Pnpla1*$^{-/-}$ epidermis.

(**a**) Altered expression profiles of genes associated with ichthyosis, lipid metabolism, keratinocyte proliferation/activation, adhesion/junction, keratinocyte differentiation, and immune response in *Pnpla1*$^{-/-}$ (KO) epidermis relative to *Pnpla*$^{+/+}$ (WT) epidermis at P0, as assessed by DNA microarray analysis. Fold changes (KO/WT) are represented by color. Equal amounts of total RNA pooled from six mice for each genotype were used. **(b)** qPCR analysis of genes for epidermal ceramide metabolism in *Pnpla1*$^{+/+}$, *Pnpla1*$^{+/-}$ and *Pnpla1*$^{-/-}$ skin at P0 (mean ± s.e.m., n = 5 animals per genotype; **P* <0.05 and ***P* <0.01 *versus Pnpla*$^{+/+}$ mice). Representative results from two independent experiments are shown.

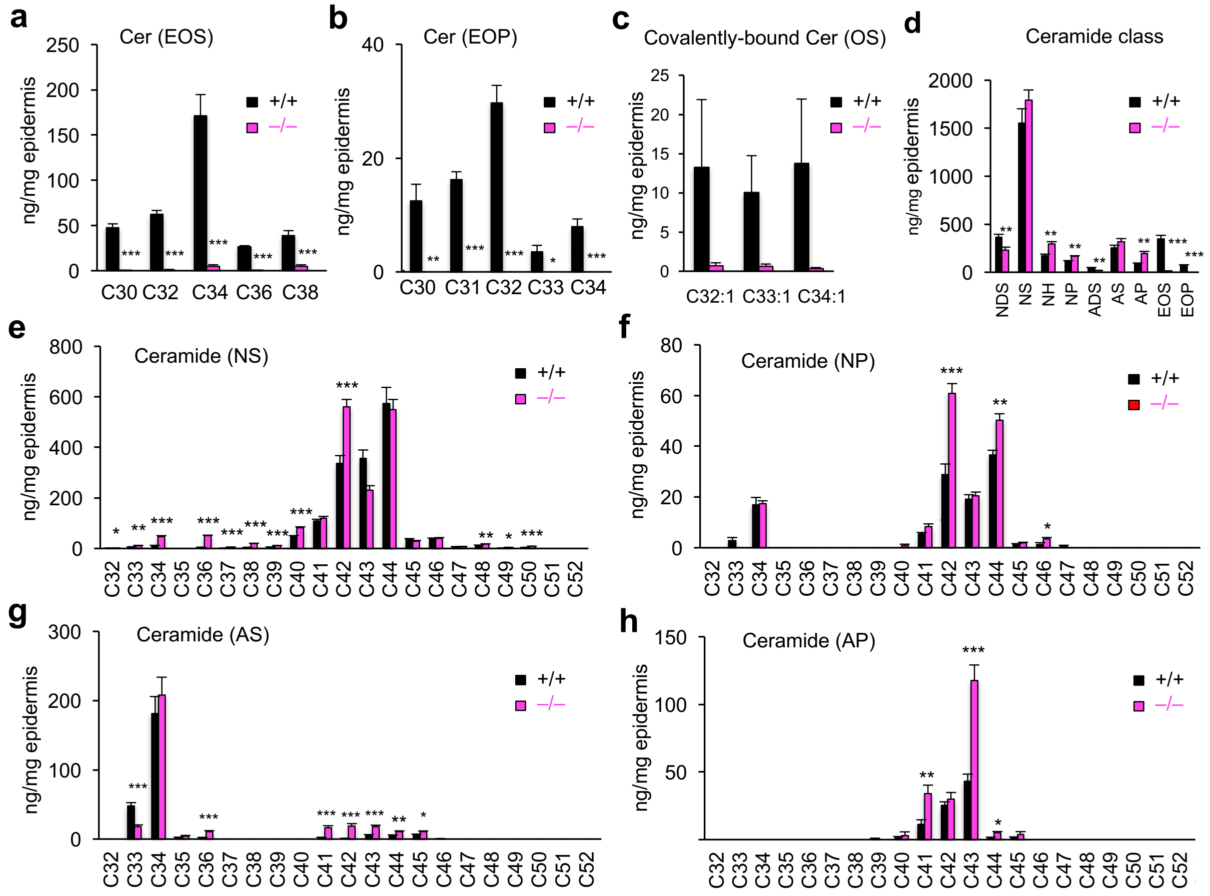

Supplementary Fig. 4. Effects of *Pnpla1* deletion on epidermal ceramides.

(a–h) LC-MS/MS quantification of EOS **(a),** EOP **(b)**, protein-bound Cer OS **(c)**, nine ceramide classes (**d**), NS **(e)**, NP **(f)**, AS **(g),** and AP **(h)** in the epidermis of *Pnpla1*$^{+/+}$ and *Pnpla1*$^{-/-}$ neonates (mean ± s.e.m., n = 5 animals; *P <0.05, **P <0.01 and **P <0.001 *versus Pnpla*$^{+/+}$ mice in an unpaired, two-tailed Student's t-test). Ceramide classes are abbreviated according to three different fatty acid components (N, non-hydroxy fatty acid; A, α-hydroxy fatty acid; EO, linoleic acid esterified ω-hydroxy fatty acid) linked via an amide bond to the sphingoid bases (S, sphingosine; DS, dehydrosphingosine; P, phytosphingosine; H, 6-hydroxysphingosine). Results are from one or two independent experiments.

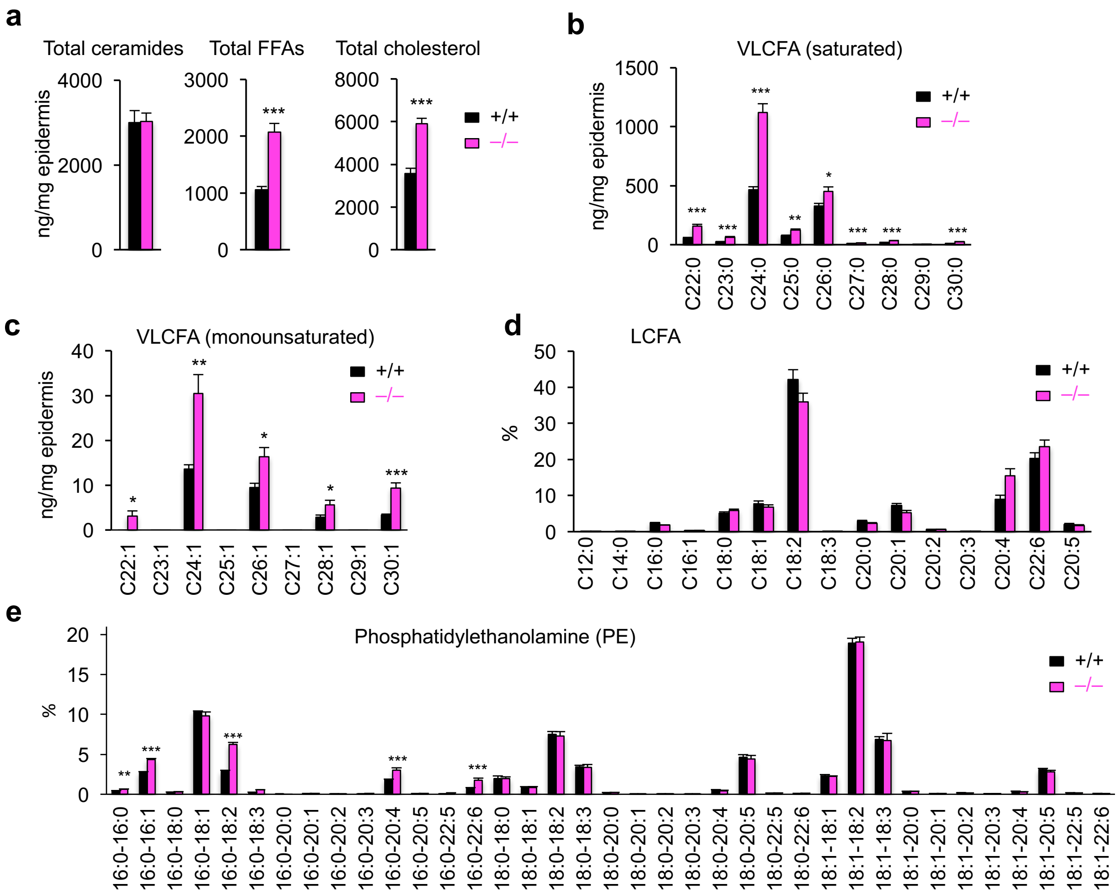

Supplementary Fig. 5. Effects of *Pnpla1* deletion on epidermal FFAs, cholesterol, and phospholipids.
(a) Quantification of total contents of ceramides, FFAs, and cholesterol in *Pnpla1*$^{+/+}$ and *Pnpla1*$^{-/-}$ neonates by LC-MS. **(b–e)** Quantification of saturated and monounsaturated forms of VLCFA **(b, c)**, LCFA **(d)**, and phosphatidylethanolamine **(e)** in the epidermis of *Pnpla1*$^{+/+}$ and *Pnpla1*$^{-/-}$ neonates by LC-MS and LC-MS/MS. Data from one or two independent experiments are shown as the mean ± s.e.m. (n = 5 animals; **P*<0.05, ***P*<0.01 and ****P*<0.001 *versus Pnpla*$^{+/+}$ mice in an unpaired, two-tailed Student's *t*-test).

Supplementary Table 1. Primers for genotyping PCR.

Primer name	Annealing temperature (°C)	Product size (bp)	5'-3' sequence
Pnpla1 WT-F	59	119	TGGCCTCTAAGCAAGTGCCCTTGT
Pnpla1 WT-R			GCAGCTTCTTGTCCCTGCACCTGA
Pnpla1 KO-F	59	330	TCTAAGGCGCATAACGATAC
Pnpla1 KO-R			TCCTCATTGTCTTAGGATGC
Cre-F	56.5	281	CCATCTGCCACCAGCCAG
Cre-R			TCGCCATCTTCCAGCAGG
Abhd5 WT-F	57	417	AGGAGGAGGTGGACTCG
Abhd5 COM-R			TTTCACAAAGTTTGCCAGG
Abhd5 KO-F	57	626	TCCTCGTGCTTTACGGTATC
Abhd5 COM-R			TTTCACAAAGTTTGCCAGG

Supplementary Table 2. Primers and UPL probes for qPCR (mouse).

Gene	Primer sequence	UPL probe
Abca12	CCTGCTAAACCAGACGATCC	#50
	ACTTGCACAAAGGGGTTCC	
Abhd5	ATCTTTGGAGCCCGATCCT	#89
	CTTCTGGCTGATCTGCATACAC	
Alox12b	CTTTGGTCCTGATGGCAAC	#105
	GACAATCAGGCCCAGGAGT	
Aloxe3	GGCCTCACTGATCTTCAACG	#4
	GTCCAGGAGACCTCGAATCTT	
Cers3	CATGATATATCTGACATTTGGCTAGAG	#38
	GTGAAGATGAAGAACAGGGTGTTA	
Cyp4f39	AAAGAAGAAAGCCAAGAGAATTGA	#89
	TGCAACAGGTAGTCGGTGAG	
Elovl4	ACGACACCGTGGAGTTCTATC	#85
	GCGGCCAGTCTGCTACAC	
Fabp5	ACGGCTTTGAGGAGTACATGA	#46
	CTCGGTTTTGACCGTGATG	
Flg	GGAGGAAGAAACACTGAGCAA	#96
	CGATGTCTTGGTCATCTGGA	
Gba	GACCAACGCTTGCTGCTAC	#34
	ACAGCAATGCCATGAACGTA	
Hbegf	TCTTCTTGTCATCGTGGGACT	#55
	CACGCCCAACTTCACTTTCT	
Hprt1	TGATAGATCCATTCCTATGACTGTAGA	#22
	AAGACATTCTTTCCAGTTAAAGTTGAG	
Krt1	TGAGCTGAAGAACATGCAAGA	#31
	CATGTAAGCTGAATCCACATCC	
Krt5	CAGAGCTGAGGAACATGCAG	#22
	CATTCTCAGCCGTGGTACG	
Krt6a	GCCAAGGCAGACAGTCTAACA	#55
	CAGGCTACGGTTGTTGTCC	
Krt6b	GGAAATTGCCACCTACAGGA	#12
	GGTGGACTGCACCACAGAG	
Lor	GGTTGCAACGGAGACAACA	#11
	CATGAGAAAGTTAAGCCCATCG	
Pnpla1	TGCGGGATTGAGATGGAG	#94
	CGCATCATCTGTACCATCTTG	
Ppard	ATGGGGGACCAGAACACAC	#11
	GGAGGAATTCTGGGAGAGGT	
Ugcg	AGTTTCAATCCAGAATGATCAGG	#4
	CATTCTGAAATTGGCTCACAAAT	

Supplementary Table 3. Primers and UPL probes for qPCR (human).

Gene	Primer sequence	UPL probe
FLG	TGCTCCCGAGAAGATCCAT	#38
	GGACTCTGAGAGGCGATCTG	
KRT1	TCCGCTTGTTGATTTCATCC	#22
	ATCAATCTCGGTTGGATTCG	
KRT5	CCACTTGGTGTCCAGAACCT	#1
	GCAGATCAAGACCCTCAACAAT	
PNPLA1	TGGTGGTTTCAGAGTTCACG	#63
	ACGAAGCAGCTGCAGTATAGG	
RPL13A	CTGGACCGTCTCAAGGTGTT	#18
	GCCCCAGATAGGCAAACTT	
